# Sex differences in treatment outcomes among U.S. service members with comorbid PTSD and MDD

**DOI:** 10.1186/s40359-025-03878-4

**Published:** 2026-01-31

**Authors:** Lisa H. Glassman, Nicholas P. Otis, Alexander C. Kline, W. Michael Hunt, Kristen H. Walter

**Affiliations:** 1 Psychological Health & Readiness Department, Naval Health Research Center, 140 Sylvester Road, , San Diego, CA 92106 USA; 2 Leidos, Inc, San Diego, CA USA; 3 Directorate of Mental Health, Naval Medical Center San Diego, San Diego, CA USA

**Keywords:** Depression, Psychotherapy, Military, Cognitive behavioral therapy, Evidence-based treatment

## Abstract

**Background:**

Posttraumatic stress disorder (PTSD) and major depressive disorder (MDD) are prevalent and deleterious conditions that commonly co-occur among service members. Identifying factors such as sex that could be linked to treatment response among service members with these conditions is critical for improving care delivery.

**Methods:**

This study examined sex differences in treatment outcomes among U.S. active duty service members with comorbid PTSD and MDD (*N* = 94; 55% women, 45% men) in a randomized controlled trial comparing behavioral activation-enhanced cognitive processing therapy (BA + CPT) and standard CPT. PTSD and MDD symptom severity was assessed at pretreatment, posttreatment, and 3-month follow-up.

**Results:**

Intent-to-treat multilevel models indicated treatment condition moderated the relationship between sex and PTSD symptoms (*p* = .020) but not depression (*p* = .16). On average, Clinician-Administered PTSD Scale for DSM-5 scores decreased significantly more among servicewomen who received CPT versus BA + CPT at posttreatment (*p* = .004) and 3-month follow-up (*p* = .049). There were no significant differences in outcomes among servicemen (*p*s > 0.05). In CPT, servicewomen reported significantly greater PTSD symptom reduction compared to servicemen at posttreatment (*p* = .039) but not at follow-up (*p* = .088). In BA + CPT, PTSD symptoms did not significantly differ between sexes at posttreatment (*p* = .054) or follow-up (*p* = .29).

**Conclusions:**

Findings suggest sex may differentially impact outcomes for CPT but not BA + CPT among service members with PTSD and MDD and could help inform shared decision-making between patients and providers.

**Trial registration:**

ClinicalTrials.gov registry; registration number NCT02874131; date of registration: 08-22-2016.

## Introduction

Posttraumatic stress disorder (PTSD) and major depressive disorder (MDD) are prevalent and deleterious conditions among service members, with approximately half of those with PTSD having a comorbid diagnosis of MDD [1]. This co-occurrence has been linked to greater symptom severity, reduced treatment effectiveness, greater treatment noncompletion, and higher suicidality compared to either diagnosis alone [2–6]. Although evidence-based treatments have proved effective for addressing symptoms of each disorder separately [7], nonresponse and dropout rates remain high [8, 9]. Emerging research has identified mechanisms of change associated with cognitive behavioral therapy (CBT) for individuals with PTSD (e.g., lower hopelessness, fear, and patient avoidance; greater negative emotionality and positive self-view) [10, 11] and MDD (e.g., reward expectation; decreases in dysfunctional beliefs) [12, 13]. Further understanding factors that influence treatment attendance and response is critical for improving care delivery, especially within the military population, who may be less responsive to evidence-based treatments [8, 9, 14, 15].

Sex may be one important factor linked to PTSD and MDD treatment attendance and outcomes. There are well-documented sex differences in the prevalence of both PTSD [16–18] and MDD [19], with higher prevalence of each condition among women compared to men. Further, women are more likely to be diagnosed with comorbid PTSD and MDD than men [18, 20]. Sex differences in the prevalences of these disorders and their comorbidity may be explained in part by research suggesting that women may be more likely than men to develop PTSD, even after exposure to a similar type of trauma (e.g., military sexual trauma, physical assault) [21, 22]. Research also indicates that sex differences in cognitive responses to trauma may influence the development of PTSD following trauma exposure. Women may be more likely to perceive a trauma as life-threatening and experience peritraumatic dissociation [23, 24] and less likely to feel self-efficacious [24] compared to men. These differences in trauma processing may have a direct effect on the development of PTSD after trauma exposure [23, 24]. Prevalence differences in the comorbidity of PTSD and/MDD likely result from an array of factors known to be affected by sex, such as treatment utilization, genetics, stigma, emotional learning, and memory processing [25–27].

There may also be sex differences in the expression of PTSD and MDD symptoms. Studies have shown that female soldiers are more likely to report a higher severity of depression symptoms compared to male soldiers [28, 29]. Furthermore, servicewomen with trauma exposure endorsed greater PTSD symptom severity than servicemen across symptom clusters, except for hypervigilance [30]. In general, women tend to report greater internalizing (e.g., anxiety) versus externalizing symptoms (e.g., anger) than do men [31]. These sex patterns in symptom severity and type have also extended to individuals with PTSD and MDD comorbidity [18, 32].

Such reported sex differences in the prevalence and expression of PTSD and MDD also suggest that these differences may exist in mental health treatment mechanisms and outcomes. In the depression literature, meta-analytic results found that sex did not moderate the effectiveness of psychotherapy outcomes in the general population [33]. In contrast, in the PTSD literature, meta-analytic findings showed that women often exhibit a better treatment response [34]. Consistent with this, in several studies specifically examining sex differences in outcomes among veterans following cognitive processing therapy (CPT), female veterans generally demonstrated greater reductions in PTSD symptomatology compared to male veterans [35, 36]. In addition to PTSD symptom improvement, women have typically reported greater improvements in quality of life, avoidance, support seeking, guilt, anger, and dissociation than men [37, 38]. Mechanisms of change in PTSD treatment may also differ between sexes. In a study of service members with PTSD who received CPT, reductions in depression were the strongest predictors of change in quality of life following treatment among women, while reductions in anger were more strongly associated with this change among men [39]. Collectively, these findings highlight the importance of examining the role of sex in treatment outcomes and processes, especially among individuals with PTSD.

Of note, most studies examining the effect of sex on treatment outcomes have included civilian or veteran samples, with minimal research involving active duty service members. Given that treatment outcomes in military populations are often attenuated compared to civilians [9] and that women represent an understudied group in military health research [40], it is particularly important to examine whether sex differences in psychological treatment outcomes exist among service members. Additionally, there are longstanding, sex-based cultural norms that exist in the military (e.g., men are stronger and more capable, women are not emotionally “tough,” military service is best suited for men) [41] that may impact treatment engagement and outcomes. A better understanding of the factors that influence service members’ response to PTSD and MDD treatments may provide a pathway to tailoring treatments and ultimately improving response within the military population.

The current study fills this gap by examining sex differences in treatment attendance, dropout, and symptom outcomes among active duty service members with comorbid MDD and PTSD. Identifying patient and intervention-related predictors of treatment response and engagement can help therapists and researchers clarify key therapeutic mechanisms, guide personalized treatment selection, and inform future intervention development. Sex differences in treatment response remains an important area for future research, within both civilian and active duty populations.

Study data were derived from a randomized clinical trial of CPT versus behavioral activation-enhanced CPT (BA + CPT) among service members with comorbid PTSD and MDD [42]. In the parent study, participants, on average, reported improvements in PTSD and MDD symptoms and diagnoses following both interventions. There were no significant differences in outcomes between treatments at posttreatment or 3-month follow-up, except for a statistically but not clinically significant difference in self-reported depression severity favoring CPT at 3-month follow-up. Importantly, the parent study included comparable numbers of males and females, helping to address the persistent challenge of the underrepresentation of women in treatment outcome research involving U.S. military populations. Aligned with literature on CPT [35, 36] and trauma-focused therapy more broadly [34], we hypothesized that servicewomen would be more likely to attend treatment compared to servicemen for both CPT and BA + CPT, given research supporting greater engagement among women with PTSD [27, 43]. Further, we predicted that servicewomen would report greater PTSD and depression symptom reduction compared to servicemen at posttreatment and 3-month follow-up. We also examined whether treatment type (i.e., CPT and BA + CPT) moderated the relationship between sex and treatment outcomes. Because BA + CPT is a novel treatment, moderation analyses were considered exploratory.

## Methods

The study protocol was approved by the Naval Medical Center San Diego Institutional Review Board in compliance with all applicable federal regulations governing the protection of human subjects. Research data were derived from approved Naval Medical Center San Diego Institutional Review Board protocol number NMCSD.2015.0039. Written informed consent was obtained from all participants involved in the study, and participation was voluntary.

### Participants

Study participants included 94 active duty service members (*n* = 52, 55% women; *n* = 42, 45% men) with comorbid PTSD and MDD who received treatment at Naval Medical Center San Diego (NMCSD), Naval Hospital Camp Pendleton, or branch clinics and were randomized to either CPT or BA + CPT as part of a clinical trial (ClinicalTrials.gov identifier NCT02874131) [42]. Inclusion criteria consisted of current PTSD and MDD diagnoses based on the Diagnostic and Statistical Manual of Mental Disorders, Fifth Edition (DSM-5) [44]. PTSD was assessed with the Clinician-Administered PTSD Scale for DSM-5 (CAPS-5) [45] and MDD was evaluated with the Structured Clinical Interview for DSM-5 Disorders, Clinical Trials Version (SCID-5-CT) [46]. Reasons for exclusion were unmanaged psychosis, suicidality with intent, past-year manic episode, substance use disorder requiring detoxification or primary treatment, and concurrent participation in trauma- or depression-focused psychotherapies.

### Measures

Participants provided demographic, service, and mental health treatment information at pretreatment. When identifying their sex, participants were asked “Are you…?” and were given two options: male or female. Attendance was measured as the number of sessions attended. Dropout was defined as attending fewer than all sessions of a full treatment protocol, consistent with the parent trial [46]. Assessment completion rates were calculated for posttreatment and 3-month follow up assessments.

Interviewer-rated PTSD and depression symptom measures for this study included the CAPS-5 and Montgomery–Åsberg Depression Rating Scale (MADRS) [47], respectively. The CAPS-5 is the gold-standard assessment for PTSD, with each item reflecting a DSM-5 criterion. Items are summed, with higher scores reflecting greater PTSD severity (range 0–80; ⍺ = 0.80). The MADRS is a validated, 10-item semi-structured interview, with items summed and higher scores indicating greater depression severity (range: 0–60; ⍺ = 0.64).

In addition, self-reported PTSD severity was assessed via the PTSD Checklist for DSM-5 (PCL-5) [48]. Each of the 20 PCL-5 items were added to create a total score (range: 0–80), with higher scores indicating greater PTSD severity. Self-reported depression severity was assessed with the 9-item Patient Health Questionnaire (PHQ-9) [49]. PHQ-9 items yield a total score (range: 0–27), with higher scores reflecting greater severity.

Assessment reliability was calculated for the CAPS-5 and MADRS. Assessments were audio recorded, with 20% selected for reliability. Interrater reliability between the assessor and fidelity rater was excellent for the MADRS (two-way mixed effects intraclass correlation coefficient [ICC] = 0.98) and good for the CAPS-5 (ICC = 0.84).

### Treatments

CPT and BA + CPT are manualized treatments that were implemented by licensed, doctoral-level psychologists who were credentialed at the participating military clinics. Out of five therapists, four were women and one was a man. Treatment consisted of weekly 60-minute individual sessions, which included 12 sessions for CPT and 14 sessions for BA + CPT. Psychologists provided treatments in both conditions. Clinical consultation was available on request. Participants who did not show to three consecutive sessions and did not respond to study staff were discontinued from treatment.

CPT is a 12-session, trauma-focused therapy that seeks to challenge and modify trauma-related beliefs and self-blame regarding the traumatic event. CPT is regarded as a first-line PTSD treatment [50] and is effective in treating PTSD among military populations. The current study used the CPT Veteran/Military Version manual [51] and included the written trauma account.

BA was selected due to its behavioral focus as a complement to the cognitive focus of CPT as well as its effectiveness in treating depression [52–54]. For this study, CPT was integrated with the Brief Behavioral Activation Treatment for Depression-Revised treatment manual [54]. This intervention is typically 5–10 sessions and designed to be flexible. BA content was emphasized in the first two sessions. The first session featured the treatment rationale, psychoeducation, and activity tracking, and the second session consisted of a value and activity assessment and behavioral goals. After these first two sessions, components of BA were integrated into CPT for the remainder of treatment. Additional information regarding BA + CPT has been published elsewhere [42, 55].

Similar to study assessments, treatment sessions were also audio recorded, and 20% of participants were randomly selected for fidelity. Therapist adherence was excellent in both treatment conditions. On average, 97% (CPT) and 94% (BA + CPT) of required content was covered at each session.

### Procedure

Health care providers at participating military treatment facilities provided study referrals. Potential participants attended a pretreatment assessment with the study assessor, who obtained voluntary, written informed consent. Consenting service members completed self-report (i.e., demographics, psychiatric symptoms, and military and treatment history information) and interview measures (i.e., MADRS, CAPS-5, SCID-5-CT) with the study assessor, who was blinded to treatment condition. If eligible, service members were then randomized to CPT or BA + CPT. Interviewer-rated symptom measures (i.e., CAPS-5, MADRS) were completed at pretreatment, posttreatment, and 3-month follow-up; self-reported symptom measures (i.e., PHQ-9, PCL-5) were completed at these time points and weekly during treatment. Additional information regarding study procedures has been published elsewhere [40, 53].

### Data analytic plan

Analyses were conducted in SPSS version 29 (IBM, Armonk, NY). Comparisons between servicewomen and servicemen on pretreatment characteristics (i.e., demographic, psychiatric, and military-related characteristics) and treatment attendance (i.e., number of sessions attended, dropout) were completed via chi-square and *t* tests. Longitudinal analyses used multilevel models to compare PTSD and depression outcomes over time (pretreatment, posttreatment, and 3-month follow-up) between servicewomen and servicemen. Analyses were intent-to-treat, with all randomized participants included in models. Restricted maximum likelihood was used in multilevel models to address missing data. Covariance matrices were compared and selected based on model fit per likelihood criteria, with consideration given to the number of specified parameters. Time was set as a repeated effect of participant using an unstructured covariance matrix. Models included time, sex, and sex × time as fixed effects. To explore the relationships among treatment condition, sex, and outcomes, fixed effects in these separate models included treatment, time, sex, and their interactions.

Male and female service members significantly differed on the following pretreatment variables: age, marital status, deployment history, combat history, pay grade (i.e., rank), time since index trauma, number of types of trauma exposure, and index trauma type (see Table 1). Given the group differences on these variables, we examined each of these pretreatment characteristics (e.g., age) in multilevel models to confirm that they were neither associated with treatment outcomes nor moderated the relationship between treatment type and outcomes. In each of these models, no two-way (e.g., age × time) or three-way (e.g., age × time × treatment) interactions were statistically significant for both PTSD and depression outcomes, indicating that these variables did not appear to be related to treatment outcomes, both overall in the full sample or differentially by treatment condition. We further confirmed these variables were not possible confounders for sex by including these characteristics as covariates in primary models. Results followed the same pattern as primary analyses when adding these characteristics to analytic models as covariates (e.g., adding age as a fixed effect in primary models, which included time, treatment, sex, and their interactions). In sum, although servicewomen and servicemen differed on several pretreatment characteristics, these variables did not appear to confound or incrementally explain the relationships between treatment type and outcomes beyond sex. Given this pattern of results and efforts to maximize power, no covariates were included in primary analyses.

**Table 1:** Sample characteristics

Characteristic^a^	Total (*N* = 94)	Women (*n* = 52)	Men (*n* = 42)	χ^2^/t
Age, *M* (*SD*)	28.5 (7.2)	25.6 (5.3)	32.0 (7.7)	−4.6***
Hispanic ethnicity, *n* (%)	21 (22.3)	15 (28.8)	6 (14.3)	2.8
Race, *n* (%)				--
American Indian or Alaska Native	3 (3.2)	3 (5.8)	0 (0)	
Asian	2 (2.1)	2 (3.8)	0 (0)	
Black or African American	14 (14.9)	5 (9.6)	9 (21.4)	
Multiracial	3 (3.2)	0 (0)	3 (7.1)	
Native Hawaiian or other Pacific Islander	4 (4.3)	1 (1.9)	3 (7.1)	
White	67 (71.3)	40 (76.9)	27 (64.3)	
Married, *n* (%)	41 (43.6)	17 (32.7)	24 (57.1)	5.6**
Years of education, *M* (*SD*)	13.4 (1.9)	13.3 (1.9)	13.5 (1.9)	−0.5
Service branch (Navy), *n* (%)	80 (85.1)	47 (90.4)	33 (78.6)	2.6
Combat history, *n* (%)	42 (44.7)	14 (26.9)	28 (66.7)	14.8***
Deployment history, *n* (%)				13.7***
0	22 (23.4)	18 (34.6)	4 (9.5)	
1	24 (25.5)	16 (30.8)	8 (19.0)	
2+	48 (51.1)	18 (34.6)	30 (71.4)	
Pay grade, *n* (%)				14.4***
E1–E4	37 (39.4)	27 (51.9)	10 (23.8)	
E5–E6	40 (42.6)	22 (42.3)	18 (42.9)	
E7–E9 or W1–O7	17 (18.1)	3 (5.8)	14 (33.3)	
Index trauma, *n* (%)				30.4***
Sexual trauma	46 (48.9)	37 (71.2)	9 (21.4)	
Combat trauma	18 (19.1)	1 (1.9)	17 (40.5)	
Other trauma	29 (30.9)	14 (26.9)	15 (35.7)	
Number of trauma types (LEC-5), *M* (SD)	8.1 (3.4)	7.3 (3.4)	9.2 (3.1)	−2.9**
Time since index trauma (months), *M* (*SD*)	77.1 (73.2)	55.0 (67.0)	105.9 (71.6)	−3.5***
Pretreatment symptom severity, *M* (*SD*)				
Interviewer-rated PTSD (CAPS-5)	45.9 (8.5)	47.4 (7.4)	44.2 (9.5)	1.8
Interviewer-rated depression (MADRS)	30.7 (7.4)	31.0 (6.5)	30.2 (8.4)	0.5
Self-reported PTSD (PCL-5)	55.0 (11.8)	59.1 (9.8)	49.8 (12.1)	4.1***
Self-reported depression (PHQ-9)	18.6 (4.0)	19.6 (3.6)	17.5 (4.2)	2.6*
Sessions attended, *M* (*SD*)	10.3 (4.9)	10.8 (4.7)	9.6 (5.1)	1.2
Treatment dropout rate, *n* (%)	36 (38.3)	18 (34.6)	18 (42.9)	0.7

*CAPS-5 *Clinician-Administered PTSD Scale for DSM-5, *E *enlisted, *O *commissioned officer, *LEC-5 *Life Events Checklist for DSM-5, *MADRS *Montgomery–Åsberg Depression Rating Scale, *PCL-5 *PTSD Checklist for DSM-5, *PHQ-9 *9-item Patient Health Questionnaire, *PTSD *posttraumatic stress disorder, *W *warrant officer

^a^ Counts vary based on available data

* *p* <.05, ** *p* <.01, *** *p* <.001

## Results

### Pretreatment comparisons

Sample characteristics are provided in Table 1, as well as comparisons between sexes on pretreatment demographic, service, and mental health characteristics. Compared to servicemen, servicewomen were younger and reported fewer deployments, lower pay grade, less time since the index trauma, and fewer types of trauma exposure. Women were more likely to endorse sexual trauma as the index event while men were more likely to report combat exposure. Men were also more likely to be married. Women and men did not differ on clinician-rated symptom severity (i.e., MADRS; CAPS-5); however, women endorsed greater self-reported depression (PHQ-9; *p* =.011, *d* = 0.54) and PTSD symptom severity (PCL-5; *p* <.001, *d* = 0.85) compared to men.

### Treatment attendance

Session attendance did not differ by sex across treatment conditions (*p* =.24). However, significant differences emerged for treatment condition within each sex. Among servicewomen, attendance did not differ (*p* =.92) between CPT (*M* = 10.9, *SD* = 4.3) and BA + CPT (*M* = 10.8, *SD* = 5.3). Among men, service members trended toward attending more BA + CPT (*M* = 11.0, *SD* = 4.5) than CPT sessions (*M* = 8.0, *SD* = 5.5), *t*(40) = 2.0, *p* =.053, *d* = 0.61). Within each treatment arm, there were trends for greater CPT attendance among women (*M* = 10.9, *SD* = 4.3) compared to men (*M* = 8.0, *SD* = 5.5), *t*(32.2) = 2.0, *p* =.057, *d* = 0.60. In BA + CPT, session attendance did not significantly differ by sex (*p* =.86). Although at trend level, men attended more sessions of BA + CPT than CPT, and in CPT, women trended toward attending more sessions than men.

Dropout rates did not significantly differ between treatment conditions (χ^2^[1, *N* = 94] = 1.6, *p* =.20) or by sex (χ^2^[1, *N* = 94] = 0.7, *p* =.41). Similar to attendance comparisons above, differences emerged for treatment condition within each sex. Servicewomen were significantly less likely to drop out of CPT (21.4%) compared to BA + CPT (50.0%), χ^2^(1, *N* = 52) = 4.7, *p* =.03, whereas dropout rates did not differ between BA + CPT (39.1%) and CPT (47.4%) among servicemen, χ^2^(1, *N* = 42) = 0.3, *p* =.59. Within CPT, there was a trend for servicewomen (21.4%) to be less likely to drop out of treatment compared to servicemen (47.4%), χ^2^(1, *N* = 47) = 3.5, *p* =.06. In contrast, within BA + CPT, dropout rates did not differ between servicewomen (50.0%) and servicemen (39.1%), χ^2^(1, *N* = 47) = 0.6, *p* =.45. Overall dropout rates did not differ significantly by treatment condition or sex; however, servicewomen were significantly less likely to drop out of CPT than BA + CPT, while dropout rates among servicemen did not differ by treatment.

Completion rates for the posttreatment and 3-month follow-up assessments did not differ between treatment conditions (*p*s > 0.33) or by sex (*p*s > 0.06). The posttreatment assessment was completed by 36 servicewomen and 36 servicemen. These numbers for the 3-month follow-up assessment were 28 and 30, respectively.

### PTSD symptoms

Estimated marginal means from multilevel models that examined outcomes for servicewomen and servicemen in each treatment condition are presented in Table 2. Clinician-rated PTSD symptoms (CAPS-5) did not significantly differ between sexes across the full sample (time × sex interaction *F* = 0.2, *p* =.80); however, the treatment × time × sex interaction was significant (*F* = 4.2, *p* =.020).

**Table 2:** Estimated marginal means from multilevel models (*N* = 94)

	Women M (95% CI)	Men M (95% CI)
BA + CPT	***n*** = **24**	***n*** **= 23**
CAPS-5		
Pretreatment	47.1 (43.7, 50.5)	43.6 (40.1, 47.1)
Posttreatment	36.5 (28.6, 44.3)	23.2 (16.5, 29.9)
3 Month	37.4 (28.3, 46.5)	27.8 (20.6, 35.0)
MADRS		
Pretreatment	31.9 (28.9, 34.9)	29.6 (26.5, 32.7)
Posttreatment	28.4 (21.9, 34.9)	19.5 (13.8, 25.2)
3 Month	26.5 (19.0, 34.0)	19.0 (13.1, 24.9)
CPT	***n*** **= 28**	***n*** **= 19**
CAPS-5		
Pretreatment	47.6 (44.4, 50.8)	44.8 (41.0, 48.7)
Posttreatment	22.5 (16.1, 28.8)	29.6 (22.1, 37.1)
3 Month	26.6 (19.6, 33.6)	33.1 (24.6, 41.7)
MADRS		
Pretreatment	30.3 (27.5, 33.0)	31.0 (27.6, 34.4)
Posttreatment	17.1 (11.8, 22.4)	20.7 (14.4, 26.9)
3 Month	17.4 (11.7, 23.0)	24.4 (17.4, 31.5)

*BA *behavioral activation, *CAPS-5 *Clinician-Administered PTSD Scale for DSM-5, *CI *confidence interval, *CPT *cognitive processing therapy, *MADRS *Montgomery–Åsberg Depression Rating Scale

**Fig. 1:**
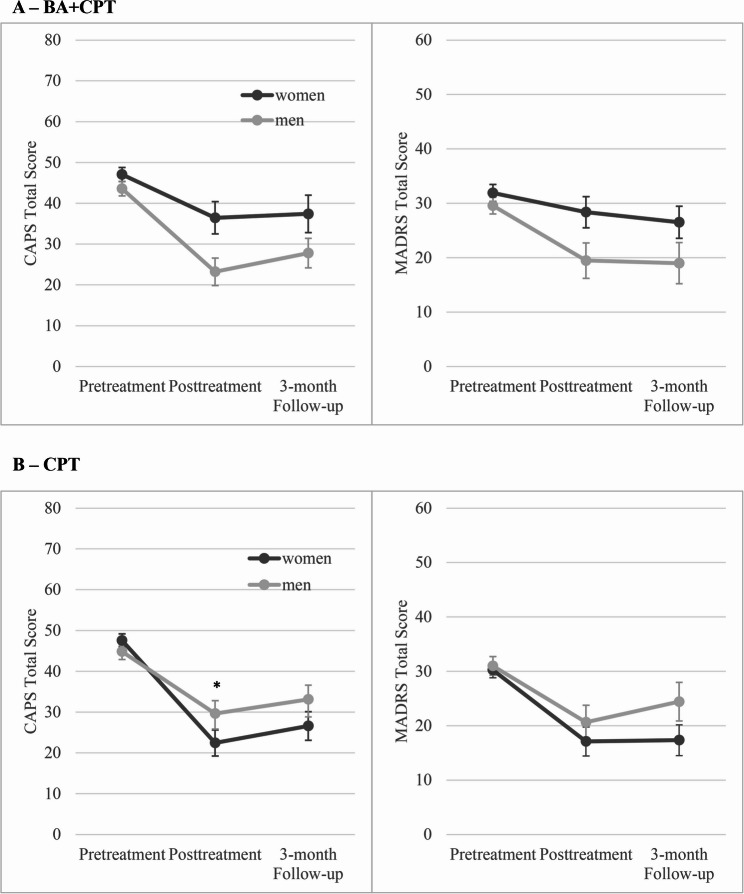
Depression and PTSD outcomes compared between servicemen and servicewomen in (**A**) BA + CPT and (**B**) CPT. BA = behavioral activation; CAPS-5 = Clinician-Administered PTSD Scale for DSM-5; CPT = cognitive processing therapy; MADRS = Montgomery–Åsberg Depression Rating Scale; PTSD = posttraumatic stress disorder **A – BA + CPT**

First, clinician-rated PTSD outcomes were compared between treatments within each sex (Fig 1). Among servicewomen, those in CPT reported significantly greater reductions in CAPS-5 scores at posttreatment (*b* = −14.5, SE = 4.9, 95% confidence interval [CI; −24.2, −4.7], *p* =.004) and 3-month follow-up (*b* = −11.3, SE = 5.6, 95% CI [−22.5, −0.1], *p* =.049) compared to those in BA + CPT. Among servicemen, there were no significant differences in symptom change on the CAPS-5 between treatments at posttreatment (*b* = 5.2, SE = 4.8, 95% CI [−4.4, 14.7], *p* =.29) or 3-month follow-up (*b* = 4.1, SE = 5.4, 95% CI [−6.7, 14.9], *p* =.45).

**Fig. 2:**
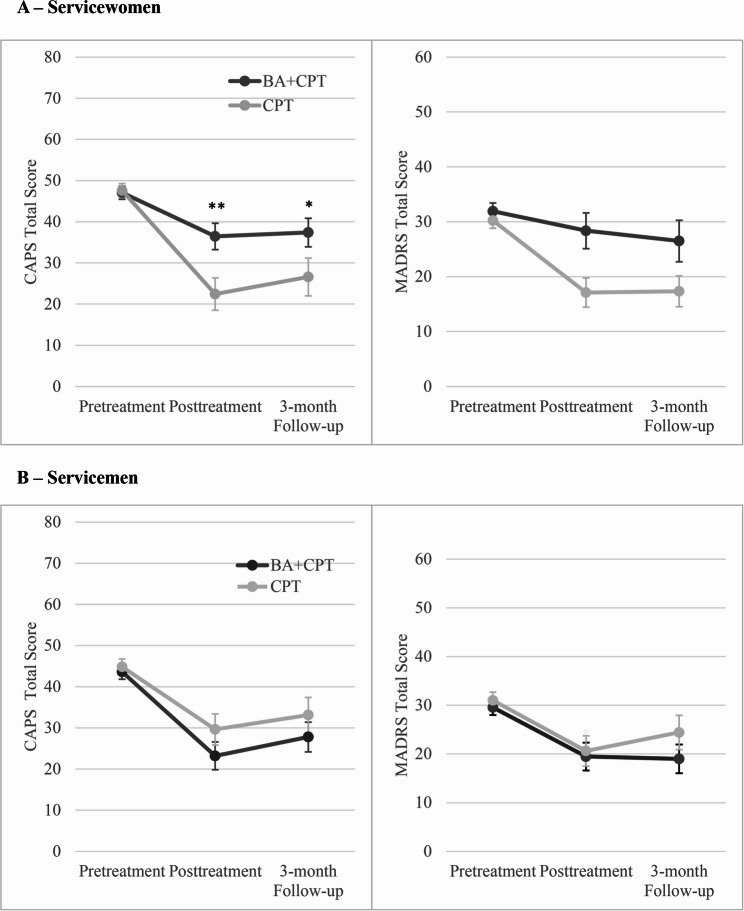
Depression and PTSD outcomes compared between BA + CPT and CPT in (**A**) servicewomen and (**B**) servicemen. BA = behavioral activation; CAPS-5 = Clinician-Administered PTSD Scale for DSM-5; CPT = cognitive processing therapy; MADRS = Montgomery–Åsberg Depression Rating Scale; PTSD = posttraumatic stress disorder

Next, the change in CAPS-5 scores was compared between sexes within each treatment (Fig 2). In CPT, women reported significantly greater reductions on the CAPS-5 at posttreatment (*b* = 9.9, SE = 4.7, 95% CI [0.5, 19.3], *p* =.039) and at a trend level at 3-month follow-up (*b* = 9.3, SE = 5.4, 95% CI [−1.4, 20.0], *p* =.088) compared to men. Conversely in BA + CPT, men trended toward greater CAPS-5 improvement at posttreatment (*b* = −9.8, SE = 5.0, 95% CI [−19.7, 0.2], *p* =.054) but not 3-month follow-up (*b* = −6.1, SE = 5.7, 95% CI [−17.5, 5.2], *p* =.29) than did women.

### Depression symptoms

Interviewer-rated depression symptom (i.e., MADRS) did not significantly differ between sexes over time (time × sex interaction *F* = 0.3, *p* =.76) or by treatment and sex over time (time × sex × treatment interaction *F* = 1.9, *p* =.16). Depression outcomes therefore did not significantly differ between servicewomen and servicemen across the entire sample or by treatment type, nor did outcomes significantly differ between treatments when comparing within each sex.

To enhance confidence in study results, we also ran identical models with self-reported PTSD (PCL-5) and depression (PHQ-9) symptom measures. These models followed a similar pattern of results, where the treatment × time × sex interaction was significant in PCL-5 models (*F* = 3.9, *p* =.026) but not PHQ-9 models (*F* = 2.5, *p* =.089).

## Discussion

Exploring sex differences in treatment outcomes is warranted given variations in the prevalence and expression of psychological conditions that often occur between men and women. This research is particularly important among military populations, who may be less responsive to evidence-based treatments [8, 9, 14, 15]. Additionally, servicewomen often report different barriers to care [56, 57] and have been underrepresented in health-focused research [40] compared to servicemen. Study results, based on data from a randomized controlled trial [42], found sex differences in treatment attendance, dropout rates, and PTSD outcomes between servicewomen and servicemen following CPT and BA + CPT for comorbid PTSD and MDD. Findings also replicate research among civilians and veterans that have found sex differences in treatment outcomes for PTSD but not MDD [33, 34].

Study findings indicated sex differences in therapy session attendance and dropout rates among service members with comorbid PTSD and MDD. Women were more likely to attend CPT sessions versus BA + CPT and were more likely to attend and complete CPT when compared to men. Although men’s attendance rates favored BA + CPT versus CPT at trend level, there were no significant differences in therapy completion rates between conditions among men. Further, women were less likely to drop out of CPT compared to BA + CPT, but there were no differences in dropout for CPT and BA + CPT among men. In CPT, women were less likely to drop compared to men at trend level. There were no differences in dropout between men and women following BA + CPT.

Our findings are in line with recent research suggesting that women are more likely to engage in treatment when it begins with cognitive work [35]. Researchers hypothesize that this may stem from early socialization and reinforcement around recognizing, expressing, and discussing thoughts and emotions; these experiences likely contribute to greater comfort with these techniques in therapy [58]. In this study, the CPT condition started with cognitive techniques while the BA + CPT condition started with behavioral activation, which may explain why women were more likely to attend CPT vs. BA + CPT sessions.

Significant sex differences emerged for both self-reported and clinician-rated PTSD symptom outcomes, highlighted by servicewomen’s response to CPT. Servicemen did not differ on PTSD outcomes between CPT and BA + CPT, suggesting that adding BA to CPT did not yield enhanced benefit for PTSD symptom improvement among men, despite their increased attendance in BA + CPT. However, results revealed significant sex differences in self-reported and clinician-rated PTSD symptom outcomes among servicewomen. Clinician-rated PTSD symptoms among servicewomen improved significantly more following CPT compared to BA + CPT at both posttreatment and 3-month follow-up. For self-reported PTSD symptoms, women who received CPT reported greater PTSD symptom reduction at posttreatment than those who received BA + CPT, and a trend level improvement at follow-up. Furthermore, a pattern emerged where servicewomen generally achieved significantly better outcomes in CPT compared to servicemen. These findings suggest that CPT may be the recommended treatment intervention for servicewomen with comorbid PTSD and MDD compared to BA + CPT. First, BA + CPT is a longer intervention and, if not significantly more effective, should be implemented with consideration of additional time and burden for the patient. Additionally, prior research, although limited, suggests that cognitive-based techniques (e.g., cognitive restructuring) may be a critical component in engaging female veterans in treatment [43]. This may explain why the inclusion of BA first, and potentially dedicating even a few minutes to check-in about BA practice assignments over the course of the protocol in lieu of exclusive attention to cognitive techniques, may have led to lower engagement and no additional benefit in outcomes for women who received BA + CPT. Future research should continue to explore the role of early cognitive techniques on treatment engagement and outcomes among women. The observed sex differences in symptom reduction following CPT raise important questions about the underlying mechanisms driving these effects. Future research is needed to disentangle whether these differences are attributable to treatment-specific processes (e.g., therapist alliance, changes in cognitive appraisals) or reflect broader differences in symptom presentation, trauma exposure, or coping styles. Identifying these mechanisms is critical for refining treatment selection and delivery to enhance outcomes across sexes.

Despite these sex differences in session attendance and PTSD symptom outcomes, we did not find sex differences in depression outcomes for clinician-rated or self-reported symptoms. Adding a depression-focused BA protocol to the beginning of CPT did not change depression outcomes based on sex. These findings mirror sex-based analyses in other populations, where the research has generally been unable to find sex differences in depression outcomes following evidence-based interventions [33].

Taken together, servicewomen and servicemen with comorbid PTSD and MDD responded differently to the two interventions. Servicewomen demonstrated a clear advantage following CPT for both session attendance and PTSD outcomes when compared to servicewomen who received BA + CPT and with servicemen who received CPT. This finding may help partly explain why research suggests that military research participants with PTSD do not respond to CPT treatment as well as civilians, as most of these studies relied on samples of primarily men [8, 9, 14]. Specifically, samples in civilian studies on PTSD treatment tend to include women with histories of interpersonal trauma whereas military studies tend to include men with combat exposure. As a result, data suggesting that military samples do not respond as well as civilian samples may be an artifact of the tendency to oversample servicemen in these studies. When interpreted considering this study’s findings, the differences seen in the larger PTSD treatment outcome literature may be, in part, a reflection of sex differences, and undersampling military populations for women, possibly reducing the effects seen for CPT. Research directly comparing military and civilian samples *within the same sex* may help to shed light on this issue and could be useful in guiding treatment recommendations.

Indeed, women have been underrepresented in military health service research [40]. Some studies have attempted to address this issue by recruiting servicewomen at the exclusion of servicemen [32] or by comparing treatment outcomes between sexes across trials [15]. Although there have been attempts to recruit both servicewomen and men, usually these studies oversample one sex, preventing sex comparisons [59, 60]. In contrast, the current study sample consisted of a comparable distribution across men and women that allowed for exploration of sex differences in treatment outcomes within the same trial. Given the lack of sex comparisons in treatment outcome research in military populations, this study offers a unique contribution by directly comparing outcomes within and across treatment protocols.

Study results should be interpreted in the context of several limitations. Findings may not generalize to other samples, as participants were active duty service members with comorbid PTSD and MDD. Women and men differed on several pretreatment characteristics (e.g., age), and it is therefore possible that a third variable (or a combination of these) may further explain the associations between sex, treatment type, and outcomes. Differences between men and women on these characteristics have been found previously in the literature [61–63] and were typical given the pragmatic nature of the parent trial. However, the pretreatment variables that differed between sexes were not significantly related to PTSD or depression outcomes, and the pattern of results in primary analyses was virtually identical when including these variables as covariates in models. Taken together, these analyses alleviate some concerns regarding a possible third confounding variable. A final limitation is that three-way interactions and the resulting comparisons were underpowered, and many results were only significant at trend level. Power analysis for the parent trial, which compared outcomes between treatment conditions rather than examining moderators of outcomes, is provided in Walter et al. (2023). Thus, study findings should be interpreted cautiously. However, given the limited research comparing sex differences in treatment outcomes among service members and the underrepresentation of servicewomen in treatment research, study results provide needed additional research in this area that ultimately may help optimize clinical care delivery.

These limitations notwithstanding, this study has notable strengths. A unique and key strength is the study sample, which included a comparable proportion of servicewomen and servicemen, which allowed for sex differences in outcomes to be examined. Related, the inclusion of servicewomen provides outcome data for this subpopulation who have been underrepresented in treatment research to date. The parent trial was a pragmatic clinical trial, where therapy was delivered by providers in real-world clinics within military treatment facilities, without participant compensation for assessments or therapy attendance [41]. The design of the trial boosts generalizability to the larger service member population. Further, the parent study used gold-standard assessments and assessed participants for up to 3 months following participation, a task that is particularly challenging within the military given the transitory nature of military service. The unique features of the trial that allowed for direct comparisons between sexes within the same trial address a significant limitation in the current treatment literature.

## Conclusions

This study supports an emerging body of literature examining sex differences in treatment outcomes, including among military populations. Study findings suggest that sex differences exist in treatment engagement and outcomes among service members with comorbid MDD and PTSD and support the use of CPT relative to BA + CPT among servicewomen in particular. Identifying sex differences may inform adaptations to evidence-based care to increase treatment engagement and outcomes. However, more research is needed in this area to improve our understanding of the nuanced differences between men and women in treatment engagement and response, including potential differences in mechanisms of change between sexes (i.e., anger, depression) [39]. Future research should aim to better understand the factors underlying sex differences before treatment adaptations and guidance can be developed. Although research on sex differences in treatment outcomes can help educate and inform patients regarding expected outcomes, it is critical that providers engage in shared decision-making that is informed, but not dictated by, this information. These findings contribute to a growing literature base supporting the investigation of sex differences in PTSD treatment outcomes in the effort to develop personalized, patient-centered care, and they highlight the importance of shared decision-making between clinicians and patients during the treatment selection process.

## Data Availability

The protocol and datasets generated and/or analyzed during the current study are not publicly available due to security protocols and privacy regulations, but they may be made available on reasonable request by the Naval Medical Center San Diego or Naval Health Research Center institutional review boards (contact phone +1.619.553.8400) or by contacting the corresponding author to facilitate the request.

## Abbreviations

BA: Behavioral activation
BA + CPT: Behavioral activation–enhanced cognitive processing therapy
CAPS: 5–Clinician–Administered PTSD Scale for DSM–5
CBT: Cognitive behavioral therapy
CPT: Cognitive processing therapy
DSM: 5–Diagnostic and Statistical Manual of Mental Disorders, Fifth Edition
MADRS: Montgomery–Åsberg Depression Rating Scale
MDD: Major depressive disorder
NMCSD: Naval Medical Center San Diego
PCL: 5–PTSD Checklist for DSM–5
PHQ: 9–Patient Health Questionnaire
PTSD: Posttraumatic stress disorder
SCID: 5–CT–Structured Clinical Interview for DSM–5 Disorders, Clinical Trials Version
